# Clinical and microbiological features of infection in alcoholic hepatitis: an international cohort study

**DOI:** 10.1007/s00535-017-1336-z

**Published:** 2017-04-07

**Authors:** Richard Parker, Gene Im, Fiona Jones, Onan Pérez Hernández, Jonathan Nahas, Aditi Kumar, Daniel Wheatley, Ashish Sinha, Emilio Gonzalez-Reimers, María Sanchez-Pérez, Antonella Ghezzi, Miruna Delia David, Christopher Corbett, Anne McCune, Guruprasad Padur Aithal, Andrew Holt, Stephen Stewart

**Affiliations:** 1 Centre for Liver Research, Institute of Biomedical Research, College of Medical and Dental Sciences, University of Birmingham, 5th Floor, Birmingham, B15 2TT UK; 2 University Hospitals Birmingham NHS Foundation Trust, Mindelsohn Way, Birmingham, B15 2TH UK; 3 Mount Sinai Medical Center, 1468 Madison Avenue, New York, NY 10029 USA; 4 Mater Misericordiae University Hospital, Eccles Street, Dublin 7, Ireland; 5 Hospital Universitario de Canarias Carretera de Ofra, 38320 San Cristóbal de La Laguna, Santa Cruz de Tenerife Spain; 6 New Cross Hospital, Royal Wolverhampton NHS Trust, Wolverhampton Rd, Heath Town, Wolverhampton, WV10 0QP UK; 7 University Hospitals Bristol NHS Foundation Trust, Marlborough Street, Bristol, BS1 3NU UK; 8 NIHR Nottingham Digestive Diseases Biomedical Research Unit, Queen′s Medical Centre, E Floor, West Block, Nottingham, NG7 2UH UK

**Keywords:** Hepatitis, alcoholic, Infection, Liver diseases, alcoholic

## Abstract

**Background:**

Previous studies have described the clinical impact of infection in alcoholic hepatitis (AH) but none have comprehensively explored the aetiopathogenesis of infection in this setting. We examined the causes, consequences and treatment of infection in a cohort of patients with AH.

**Methods:**

We undertook a retrospective cohort study of patients with AH admitted between 2009 and 2014 to seven centres in Europe and the USA. Clinical and microbiological data were extracted from medical records. Survival was analysed with Kaplan–Meier analysis and Cox proportional hazards analysis to control the data for competing factors. Propensity score matching was used to examine the efficacy of prophylactic antibiotics administered in the absence of infection.

**Results:**

We identified 404 patients with AH. Of these, 199 (49%) showed clinical or culture evidence of infection. Gut commensal bacteria, particularly *Escherichia coli* and *Enterobacter* species, were most commonly isolated in culture. Fungal infection was rarely seen. Cultured organisms and antibiotic resistance differed markedly between centres. Infection was an independent risk factor for death (hazard ratio for death at 90 days 2.33, 95% confidence interval 1.63–3.35, *p* < 0.001). Initiation of antibiotic therapy on admission in the absence of infection did not reduce mortality or alter the incidence of subsequent infections. Corticosteroid use increased the incidence of infection but this did not impact on survival.

**Conclusions:**

In this large real-world cohort of patients with AH, infection was common and was associated with reduced short-term survival. Gram-negative, gut commensal bacteria were the predominant infective organisms, consistent with increased translocation of gut bacteria in AH; however, the characteristics of infection differ between centres. Infection should be actively sought and treated, but we saw no benefits of prophylactic antibiotics.

**Electronic supplementary material:**

The online version of this article (doi:10.1007/s00535-017-1336-z) contains supplementary material, which is available to authorized users.

## Introduction

Alcoholic liver disease (ALD) accounts for significant morbidity and mortality globally, causing 14.5 million disability-adjusted life-years and nearly 500,000 deaths in 2010 [1]. Alcoholic hepatitis (AH), typified by jaundice and coagulopathy [2], is the most florid manifestation of ALD [2]. Registry data from Denmark show an increasing incidence of AH from 1999 to 2008, particularly in women and older patients [3]. Severe AH is associated with a high 1-month mortality of approximately 15% [4, 5]. Despite decades of research, no pharmacotherapies have been shown to be unequivocally effective. [5, 6] Liver transplant is controversial, but has shown benefit in highly selected cases [7]. Most recently the largest randomised trial in AH to date failed to show a categorical benefit for corticosteroids, describing a non-statistically significant reduction in mortality in patients treated with steroids at 1 month countered by increased mortality at 6 months due to increased rates of sepsis in the group receiving steroid therapy [5].

Infections occur frequently in AH. Bacterial infection occurs in approximately half of patients admitted to hospital with AH [8–13], with a recent study showing that 64% of patients had detectable signs of sepsis [13]. The high incidence of infection is due to several factors: defects in immune cell function in AH may predispose to infection [14, 15], whereas changes in gut permeability and dysbiosis result in translocation of gut-resident bacteria and endotoxin [16–18]. Moreover, secondary mechanical effects on respiratory function such as atelectasis due to abdominal ascites or hydrothorax predispose patients to pulmonary infection.

Infection in AH is associated with high mortality [8, 19, 20]. This is particularly concerning given the increased risk of infection with corticosteroid treatment, although an important study by Louvet et al. [21], showed corticosteroid use to be safe once infection had been controlled. Many centres recommend the use of empirical antibiotics [22]. This is not currently supported by evidence, but trials are under way to investigate the efficacy of this approach [23, 24]. Many trials have reported the overall incidence of infection but data regarding the underlying causes of infection are available only from smaller studies [8]. This study examined a large, international cohort of patients with AH to describe the incidence, distribution and causes of infection in AH, the clinical consequences and the effect of treatment with antibiotics and corticosteroids.

## Methods

We studied a cohort of patients with AH admitted to our institutions (University Hospitals Birmingham NHS Foundation Trust, Bristol Royal Infirmary, Nottingham University Hospitals NHS Trust and New Cross Hospital, Royal Wolverhampton NHS Trust, all in the UK; Hospital Universitario de Canarias, San Cristóbal de La Laguna, Spain; Mater Misericordiae University Hospital, Dublin, Ireland; Mount Sinai Medical Center, New York, USA) between 2012 and 2014. Cases were identified from prospectively maintained databases in some centres (Nottingham, Bristol, San Cristóbal de La Laguna) or identified retrospectively from hospital records with use of International Statistical Classification of Diseases and Related Health Problems tenth revision (ICD-10) codes K70.1, K70.4 and K70.9. For retrospectively identified cases, the diagnosis of AH was confirmed by careful review of clinical and laboratory records. Patients were included if there was a history of recent excessive alcohol intake (guidelines in various countries are summarised in Table S1), biochemistry findings consistent with AH (bilirubin concentration greater than 150 mmol/L, with aspartate aminotransferase and/or alanine aminotransferase concentration less than 300 IU/mL [2]) and no evidence of other forms of liver disease in the history on from blood tests for viral infection or metabolic disease. Liver biopsy was not required for inclusion. Patients with a history of AH in the preceding 6 months were excluded. Data were collected regarding patient characteristics, biochemistry, haematology, survival and antimicrobial use. Missing data were not imputed. Patients were treated according to local protocols without any study-specific procedures. Corticosteroid or pentoxifylline use was at the discretion of the treating clinicians. Where corticosteroids were prescribed, 40 mg prednisolone daily was used, and response was gauged over 7 days of treatment as per the Lille model [25]. As no study-specific procedures were performed and only routinely collected data were reviewed, we sought neither individual patient consent nor specific research ethics approval. Applicable institutional approvals are noted in the electronic supplementary material.

The occurrence of infection at any point during hospital admission was noted. Infection was defined as per previous investigators [25, 26]:

1. Spontaneous bacterial peritonitis: ascitic fluid polymorphonuclear cell count greater than 250/µL with or without positive fluid culture findings
2. Lower respiratory tract infection: new pulmonary infiltrate on chest radiograph
3. Bacterial gastroenteritis: diarrhoea or dysentery with positive stool culture findings
4. *Clostridium difficile* colitis: diarrhoea with positive *C. difficile* toxin detection
5. Urinary tract infection: positive urine dipstick result for leukocytes or nitrites, or urine white blood cell count greater than 15 per high-power field with positive urine culture findings in a symptomatic patient
6. Occult bacteraemia: positive blood culture findings without a source of infection.

Fever and leucocytosis are classically associated with AH, and accordingly these factors were not considered prima facie evidence of infection. Similarly bacteroascites in the absence of an ascitic fluid polymorphonuclear cell count greater than 250/µL was not considered to be de facto evidence of infection.

Groups were compared with Student’s *t* test for normally distributed data or the Mann–Whitney test if data were not normally distributed. Fisher’s exact test was used for categorical data. Binary logistic regression to generate odds ratios was used to examine factors predisposing to infection. Survival was analysed with Kaplan–Meier curves and Cox proportional hazards ratios calculated to control the data for confounding factors. Data were censored after 12 months of follow-up. Propensity score matching was used to investigate prophylactic antibiotic use, with a match tolerance of 0.2 with a preference for exact matches. Statistical analysis was performed with IBM SPSS Statistics version 23 (IBM, Armonk, NY, USA).

## Results

We identified 404 patients admitted to our institutions with AH. The characteristics of the included patients are shown in Table 1. Most of the patients were male and middle-aged, consistent with recent participants in trials in AH [5, 27]. Of these, 199 patients (49%) showed evidence of infection. To consider the impact of variations in patients and practice between our centres, we analysed differences in the severity of disease (assessed with the discriminant function), survival and prednisolone and antibiotic use between our centres to inform subsequent analysis. The severity of disease (one-way ANOVA *p* = 0.02), prednisolone use (ANOVA *p* < 0.01) and antibiotic use (ANOVA *p* < 0.01) differed significantly between centres. Survival did not differ by centre (log-rank *p* = 0.207). The incidence of infection did not differ significantly between centres (chi–squared *p* = 0.10).

**Table 1:** Characteristics of included patients

	Entire cohort (*n* = 404)		No evidence of infection (*n* = 205)		Infection (*n* = 199)		*p* (no infection vs infection)
	Average	Variance	Average	Variance	Average	Variance	
Age (years)	49.0	57	49.0	56	48.0	50	0.779
Prothrombin time (s)	28.5	11.7	29.6	10.0	29.5	13.2	0.101
Bilirubin (mmol/L)	330	182.1	320	176	341	187	0.241
Creatinine (mmol/L)	131	125	113	107	149	138	0.004
Sodium (mmol/L)	132.47	7.51	132	7.1	133	7.9	0.503
Urea (mmol/L)	24.28	35.32	20.7	29.9	28.0	39.8	0.031
Albumin (g/dL)	26.9	6.4	27.7	6.6	26.1	6.2	0.016
White blood cell count (×10^9^/L)	13.53	9.42	12.51	8.9	14.6	9.9	0.028
Platelet count (×10^9^/mL)	128.8	86.5	130.2	87.6	127	85.6	0.572
Discriminant function	106.3	113.2	91.8	75	121	140	0.009
Prednisolone use	156 (39%)		78 (39%)		78 (39%)		0.340

Data are shown as the number and percentage of patients for prednisolone use or as the mean and variance compared with the Mann–Whitney test

Patients with evidence of infection had higher creatinine concentration (148 mg/dL vs 113 mg/dL, *p* = 0.004), higher white blood cell count (14.5 × 10^9^/L vs 12.5 × 10^9^/L, *p* = 0.028), lower albumin concentration (2.61 g/dL vs 2.77 g/dL, *p* = 0.016) and severer disease as assessed by the discriminant function (121 vs 92, *p* = 0.009). In binary regression analysis, higher creatinine concentration (odds ratio 1.004, 95% confidence interval 1.001–1.007, *p* = 0.016) and lower albumin concentration (odds ratio 0.953, 95% confidence interval 0.916–0.991, *p* = 0.016) were the only factors independently associated with infection (Table S2).

**Fig. 1:**
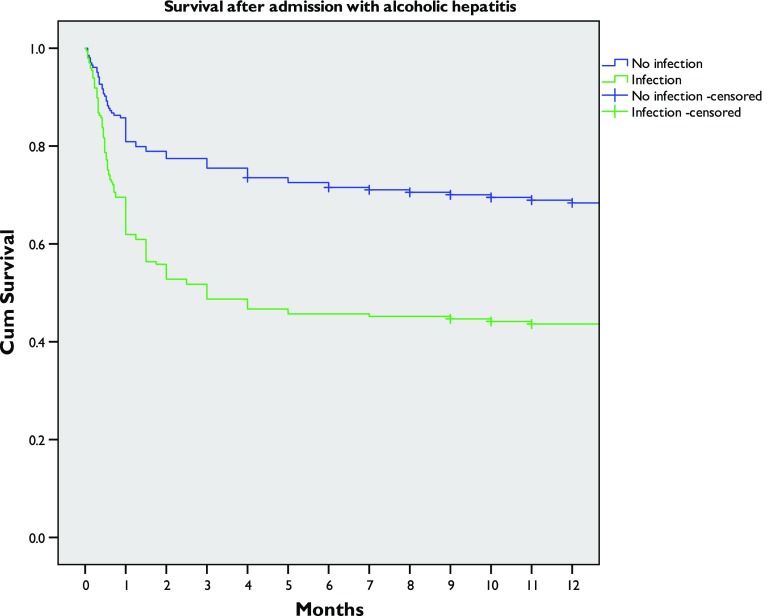
Survival of patients after admission with alcoholic hepatitis with or without evidence of infection. Log-rank *p* < 0.001

The presence of infection was associated with worse survival by Kaplan–Meier analysis (log-rank *p* < 0.001) (Fig. 1). When other factors (age, bilirubin concentration, creatinine concentration, prothrombin time, albumin concentration, white blood cell count and centre) were controlled for by Cox proportional hazards analysis, infection remained a significant risk factor for death at 28 days (hazard ratio 1.92, 95% confidence interval 1.25–2.94, *p* = 0.002), 90 days (hazard ratio 2.33, 95% confidence interval 1.63–3.35, *p* < 0.001) and 12 months (hazard ratio 2.01, 95% confidence interval 1.41–2.84, *p* < 0.001) after admission (Table S3, Fig. S1).

Of the 199 infections, 71 (36%) were evident at the time of presentation to hospital and 128 (64%) developed during admission. Infection was an early phenomenon, with 50% of infections occurring within 24 h of admission and 73% of infections occurring within 1 week of admission (Fig. S2). No significant difference in survival was seen between patients with infection on admission and those developing infection during admission (log-rank *p* = 0.169).

### Site and causes of infection

The commonest sites of infection were the chest in 80 patients (40%), the urinary tract in 69 patients (35%), the peritoneum in 28 patients (14%) and blood in 21 patients (11%) (Table 2). Five patients had microbiological evidence of infection but outside the defined criteria: three patients had bacteroascites—organisms grown on culture of ascitic fluid, but without evidence of spontaneous bacterial peritonitis by the cell count criterion. In addition, one patient had oesophageal candidiasis, one patient grew *Candida albicans* from bile and one patient had PCR evidence of *C. difficile* in stool without prior evidence of infection. Short-term and medium-term mortality were similar between all sites of infection (Fig. S3), but 12-month survival was worse in patients with lower respiratory tract infection and bloodstream infection compared with those with urinary tract infection or spontaneous bacterial peritonitis.

**Table 2:** Sites of infection and cultured organisms (excluding a single patient with positive *Clostridium difficile* PCR without prior infection)

Site of infection	Number of infections	Culture negative	Culture positive		
			Number	Species	Number according to species
Chest	80	56	24	*Escherichia coli*	1
				*Enterobacter* species	9
				*Staphylococcus aureus*	5
				*Corynebacterium*	1
				*Neisseria polyscchaerae*	1
				*Streptococcus pneumoniae*	4
				*Lactobacillus minutus*	1
				*Klebsiella*	2
Urinary tract	69	18	51	*Escherichia coli*	24
				*Klebsiella* species	9
				*Enterococcus* species	10
				*Candida* species	2
				Mixed growth	2
				*Morganella morganii*	1
				*Actinobacter baumannii*	1
				*Stenotrophomonas maltophilia*	1
				*Proteus mirabilis*	1
peritoneum (i.e., ascitic culture)	28	18	10	*Escherichia coli*	1
				*Klebsiella* species	2
				*Eggerthella lenta*	1
				Methicillin-resistant *Staphylococcus aureus*	1
				*Pseudomonas aeruginosa*	1
				*Enterobacter* species	3
				*Candida albicans*	1
Blood	21	0	21	*Escherichia coli*	3
				*Enterobacter* species	4
				*Corynebacterium*	1
				*Haemophilus influenzae*	1
				*Streptococcus* species	4
				*Klebsiella*	1
				*Staphylococcus* species	6
				*Actinobacter baumanii*	1
Total	199	92	106		

Organisms were grown from culture from 106 patients with infections (53% of all infections). Cultured organisms are listed in Table 2. Overall, there was a predominance of Gram-negative bacteria, accounting for 67% of isolated organisms, and *Enterobacteriaceae* in particular. *Escherichia coli* was the most commonly isolated single organism, found in 29 patients (27% of all isolated organisms), and *Enterobacter* species were grown in culture from 26 patients (25% of all infections). Gram-positive bacteria were less commonly seen (19 cultures, 18% of all positive cultures). There was no particular relationship between the type of isolated organism and the site of infection. *Candida* was grown in culture from three patients (3% of all positive culture results), of whom two died during follow-up, both within 3 months of admission; however the overall the type of organism grown in culture was not associated with survival (Fig. S4).

**Fig. 2:**
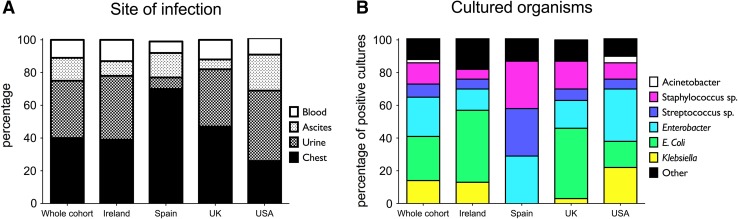
**a** Site and **b** cause of infection by geographical location of the centres

The site and cause of infection differed significantly between geographical locations of the centres (Fig. 2). Multiresistant organisms were seen in 24 cultures (22.6% of all cultures). The most commonly seen was vancomycin-resistant enterococcus (13 cultures), and also observed were methicillin-resistant *Staphylococcus aureus* (cultured from five patients), carbapenem-resistant enterococci (three cultures) and organisms producing extended-spectrum *β*-lactamases (four cultures). The presence of resistant organisms did not appear to have an adverse effect on survival (Supplementary Fig. 4).

Three blood cultures grew organisms that were considered potential contaminants: two coagulase-negative staphylococci and one *Streptococcus milleri*. All three patients died within 12 months of admission, with a median survival of 4 months. Removal of these individuals with potential contaminants from univariate Kaplan–Meier survival analysis did not significantly change the results (log-rank *p* < 0.001). Ten cases of *C. difficile* infection were observed, nine in patients who had previously been treated for infection, and one in a patient without evidence of prior infection. All patients had received antibiotics before the diagnosis of *C. difficile* infection. The presence of *C. difficile* did not appear to have an adverse effect on survival: only two of this subgroup died during follow-up.

### Antimicrobial therapy

Antimicrobial use was common: overall, 274 patients (68%) were prescribed antibiotics, and 76 patients (19%) were prescribed antifungal treatment. To examine the efficacy of prophylactic antibiotics to reduce infection and improve survival, the subgroup of patients without infection at admission was studied (Fig. S4), including 301 patients without evidence of infection in the first 48 h of their admission. Prophylactic antibiotic use was defined as antibiotic treatment within 24 h of admission. No survival benefit was seen in univariate Kaplan–Meier analysis. Indeed an apparent deficit in survival was seen with antibiotic use (log rank *p* = 0.04); however, there were significant differences between groups with regard to creatinine concentration and albumin concentration (Table S4).

**Fig. 3:**
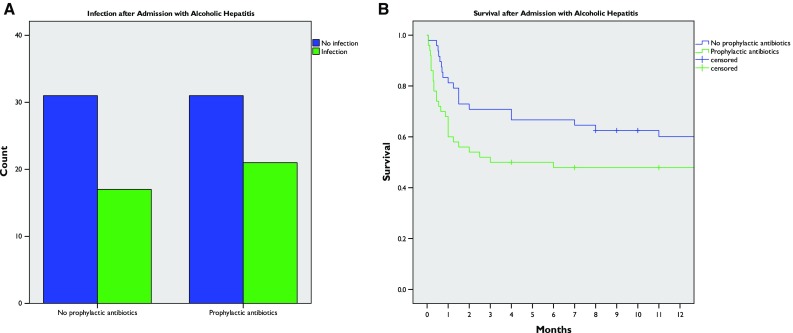
Efficacy of prophylactic antibiotic therapy: **a** incidence of infection with or without prophylactic antibiotics; **b** overall survival with or without prophylactic antibiotics

To address these differences and overcome some of the difficulties of retrospective analysis, propensity score matching was used to match equivalent patients who received or did not receive prophylactic antibiotics. This identified 50 patients given prophylactic antibtiotics matched on a 1:1 basis with controls. This process removed any significant differences between groups (Table 3). No reduction in overall infection rate was seen (40% vs 35% in the prophylactic group and the non-prophylactic group respectively, chi-squared *p* = 0.682) (Fig. 3a). Infections occurred later in patients given prophylactic antibiotics (median 10 days after admission vs 7 days in controls) but this did not reach statistical significance (Mann–Whitney test *p* = 0.310). Overall survival did not differ significantly between the groups (Fig. 3b) (log-rank *p* = 0.051).

**Table 3:** Characteristics of patients with and without prophylactic antibiotic therapy after propensity score matching

	Total		No prophylactic antibiotics		Prophylactic antibiotics		*p*
	Mean	Variance	Mean	Variance	Mean	Variance	
Age (years)	49.2	10.0	49.9	9.1	48.6	10.7	0.510
INR	2.2	1.5	2.3	1.5	2.2	1.4	0.791
Creatinine (mmol/L)	133.6	128.9	114.2	119.6	151.5	135.6	0.150
Sodium (mmol/L)	130.0	7.4	130.9	6.2	129.2	8.3	0.266
Urea (mmol/L)	17.5	25.1	20.1	24.0	15.2	26.0	0.333
Albumin (g/dl)	24.0	6.0	25.2	6.0	24.1	6.1	0.833
White blood cell count (×10^9^/L)	11.9	7.2	11.3	6.9	12.6	7.5	0.377
Platelet count (×10^9^/L)	122.4	80.7	117.9	85.3	126.5	77.0	0.597

**INR** international normalized ratio

### Corticosteroid use and infection

Overall, corticosteroids were prescribed in 156 patients (39%) of the entire cohort. The same proportion of patients received corticosteroids in non-infected and infected groups (Table 1). After exclusion of patients with evidence of infection at admission (total *n* = 332), we observed a statistically significant increase in the overall incidence of infection with corticosteroid treatment (70% in corticosteroid-treated patients vs 47% in non-treated patients, Fisher’s exact test *p* = 0.027). For survival analysis, only patients receiving corticosteroid therapy for more than 3 days were included (*n* = 147). Despite the increase in the incidence of infections, a small but statistically significant survival benefit of corticosteroids was observed (hazard ratio 0.626, 95% confidence interval 0.405–0.969, *p* = 0.036) at 28 days after admission.

## Discussion

These detailed observations of 404 patients with AH from centres in Europe and North America confirm that infection is common in AH, seen in nearly half of patients, and is associated with worse outcomes. Most infections seen in AH are lower respiratory tract or urinary tract infections, and are usually caused by Gram-negative gut commensal bacteria. However, within the limits of this retrospective study, we observed that the use of antimicrobials early in admission delayed but did not reduce the occurrence of infections, without an effect on mortality.

This large, multinational study provides a greater level of detail regarding infectious events in AH than has been reported to date. We confirm that gut or urinary tract commensal bacteria, particularly *E. coli*, are commonly implicated in infections in AH. In contrast to a recent report from Belgium, we did not find any cases of aspergillosis [28], and we found only a few cases of candidias. Importantly, the underlying pathogens differ between geographical locations and different units—for example, the resistance patterns differed between centres in this study, as did the incidence of *C. difficile* infection. This emphasises the importance of local microbiological expertise and protocols. Our retrospective data have limitations. Screening for infection was not systematic and depended on the practice of individual clinicians. Similarly, there was no preagreed treatment protocol for treating infection when it was detected. The use of prophylactic antibiotics (i.e. in the absence of hard evidence of infection) was common, and may reflect the severity of disease that we were unable to control for despite multivariate analysis.

This cohort describes a ‘real-life’ cohort, which will differ from selected trial populations. Perhaps the most important difference is that we defined AH on clinical grounds rather than relying on biopsy. There is no consensus regarding the necessity of biopsy [29], and recently the NIAAA Alcoholic Hepatitis Consortia recommended a biopsy for inclusion in studies only when doubt exists regarding diagnosis [30]. It is notable that in the recent STOPAH trial only a minority of patients underwent biopsy [5]. Nevertheless, this may explain some differences between our data and data from recent trials. In particular, the overall incidence of infection in the STOPAH trial was only 20% [5], much lower than the rates seen in our cohort. Another large study, by Nguyen-Khac et al. [28], also described a lower incidence of infection of approximately 30% over 6 months. In both trials, patients were excluded from both trials for persistent infection at the baseline, which may explain the differing observations. In common with the STOPAH trial, we saw an increased risk of infection with steroid use, but in contrast to the findings of Louvet et al. [21], we did not observe a negative impact of corticosteroids on survival in the presence of infection. This latter point may be confounded by clinicians being reluctant to use corticosteroids in patients with signs of severe infection, a weakness of a retrospective approach.

Clinicians caring for individuals with AH should be aware of the high risk of infection, and the increased mortality associated with infection, particularly if it is pulmonary or blood-borne. Systematic screening for infection should be part of routine management of such patients. The data presented here regarding likely causative organisms can guide empirical therapy before cultures results are available. Other measures such as management of ascites, hydrothorax and nutritional therapy to improve gut permeability are important. Although retrospective data should be interpreted with caution, our data do not suggest a benefit of antibiotic use in the absence of infection.

In summary, our data confirm the high burden of infection in patients with AH. The early use of antibiotics in high-risk patients to prevent subsequent infections or improve survival is not supported. This will be clarified by prospective clinical trials currently under way.

## Electronic supplementary material

Below is the link to the electronic supplementary material.

Supplementary material 1 (PDF 4978 kb)

## References

[1] Rehm J, Samokhvalov AV, Shield KD (2013). Global burden of alcoholic liver diseases. J Hepatol.

[2] Lucey MR, Mathurin P, Morgan TR (2009). Alcoholic hepatitis. N Engl J Med.

[3] Sandahl TD, Jepsen P, Thomsen KL (2011). Incidence and mortality of alcoholic hepatitis in Denmark 1999–2008: a nationwide population based cohort study. J Hepatol.

[4] Mathurin P, Mendenhall CL, Carithers RL (2002). Corticosteroids improve short-term survival in patients with severe alcoholic hepatitis (AH): individual data analysis of the last three randomized placebo controlled double blind trials of corticosteroids in severe AH. J Hepatol.

[5] Thursz MR, Richardson P, Allison M (2015). Prednisolone or pentoxifylline for alcoholic hepatitis. N Engl J Med.

[6] Singh S, Murad MH, Chandar AK (2015). Comparative effectiveness of pharmacological interventions for severe alcoholic hepatitis: a systematic review and network meta-analysis. Gastroenterology.

[7] Mathurin P, Moreno C, Samuel D (2011). Early liver transplantation for severe alcoholic hepatitis. N Engl J Med.

[8] Verma S, Ajudia K, Mendler M (2006). Prevalence of septic events, type 1 hepatorenal syndrome, and mortality in severe alcoholic hepatitis and utility of discriminant function and meld score in predicting these adverse events. Dig Dis Sci.

[9] Louvet A, Diaz E, Dharancy S (2008). Early switch to pentoxifylline in patients with severe alcoholic hepatitis is inefficient in non-responders to corticosteroids. J Hepatol.

[10] Altamirano J, Fagundes C, Dominguez M (2012). Acute kidney injury is an early predictor of mortality for patients with alcoholic hepatitis. Clin Gastroenterol Hepatol.

[11] Potts JR, Goubet S, Heneghan MA (2013). Determinants of long-term outcome in severe alcoholic hepatitis. Aliment Pharmacol Ther.

[12] Altamirano J, Miquel R, Katoonizadeh A (2014). A histologic scoring system for prognosis of patients with alcoholic hepatitis. Gastroenterology.

[13] Michelena J, Altamirano J, Abraldes JG (2015). Systemic inflammatory response and serum lipopolysaccharide levels predict multiple organ failure and death in alcoholic hepatitis. Hepatology.

[14] Feliu E, Gougerot MA, Hakim J (1977). Blood polymorphonuclear dysfunction in patients with alcoholic cirrhosis. Eur J Clin Investig.

[15] Mookerjee RP, Stadlbauer V, Lidder S (2007). Neutrophil dysfunction in alcoholic hepatitis superimposed on cirrhosis is reversible and predicts the outcome. Hepatology.

[16] Qin N, Yang F, Li A (2014). Alterations of the human gut microbiome in liver cirrhosis. Natur..

[17] Rao R (2009). Endotoxemia and gut barrier dysfunction in alcoholic liver disease. Hepatology.

[18] Bala S, Marcos M, Gattu A (2014). Acute binge drinking increases serum endotoxin and bacterial DNA levels in healthy individuals. PLoS One.

[19] Coffin PO, Sharpe BA (2007). Cause of death in alcoholic hepatitis. J Hosp Med.

[20] Yu CH, Xu CF, Ye H (2010). Early mortality of alcoholic hepatitis: a review of data from placebo-controlled clinical trials. World J Gastroenterol.

[21] Louvet A, Wartel F, Castel H (2009). Infection in patients with severe alcoholic hepatitis treated with steroids: early response to therapy is the key factor. Gastroenterology.

[22] Mandrekar P, Bataller R, Tsukamoto H (2016). Alcoholic hepatitis: translational approaches to develop targeted therapies. Hepatology.

[23] ClinicalTrials.gov. Randomised open-label multicenter study evaluating ciprofloxacin in severe alcoholic hepatitis. ClinicalTrials.gov identifier: NCT02326103. http://clinicaltrials.gov/ct2/show/NCT02326103?term=alcoholic+hepatitis&rank=18 (2016). Accessed 30 Jun 2016.

[24] ClinicalTrials.gov. Efficacy of antibiotic therapy in severe alcoholic hepatitis treated with prednisolone (antibiocor). ClinicalTrials.gov identifier: NCT02281929.https://clinicaltrials.gov/ct2/show/NCT02281929?term=alcoholic+hepatitis&cntry1=EU%3AFR&rank=2 (2016).

[25] Louvet A, Naveau S, Abdelnour M (2007). The lille model: a new tool for therapeutic strategy in patients with severe alcoholic hepatitis treated with steroids. Hepatology.

[26] Cazzaniga M, Dionigi E, Gobbo G (2009). The systemic inflammatory response syndrome in cirrhotic patients: relationship with their in-hospital outcome. J Hepatol.

[27] Bajaj JS, O’leary JG, Reddy KR (2012). Second infections independently increase mortality in hospitalized patients with cirrhosis: the North American Consortium for the Study of End Stage Liver Disease (NACSELD) experience. Hepatology.

[28] Nguyen-Khac E, Thevenot T, Piquet MA (2011). Glucocorticoids plus N-acetylcysteine in severe alcoholic hepatitis. N Engl J Med.

[29] Gustot T, Maillart E, Bocci M (2014). Invasive aspergillosis in patients with severe alcoholic hepatitis. J Hepatol.

[30] Stevenson M, Lloyd-Jones M, Morgan M (2012). Non-invasive diagnostic assessment tools for the detection of liver fibrosis in patients with suspected alcohol-related liver disease: a systematic review and economic evaluation. Health Technol Assess.

[31] Crabb DW, Bataller R, Chalasani NP (2016). Standard definitions and common data elements for clinical trials in patients with alcoholic hepatitis: recommendation from the NIAAA Alcoholic Hepatitis Consortia. Gastroenterology.

