# Supplementary material for: Clinical and microbiological features of infection in alcoholic hepatitis: an international cohort study
Source: J Gastroenterol. 2017 Apr 7;52(11):1192–200. doi: 10.1007/s00535-017-1336-z (PMC5666044; doi:10.1007/s00535-017-1336-z)
Supplement: Supplementary file 1 — Supplementary material 1 (PDF 4978 kb) [file 535_2017_1336_MOESM1_ESM.pdf]

Redundant or Duplicate Publication

When submitting a paper, an author should always make a full statement to the editor about all submissions and previous reports that might be regarded as redundant or duplicate publication of the same or very similar work. The author should alert the editor if the work includes subjects about whom a previous report has been published. Any such work should be referred to and referenced in the new paper. Copies of such material should be included with the submitted paper to help the editor decide how to deal with the matter.

If redundant or duplicate publication is attempted or occurs without such notification, authors should expect editorial action to be taken. At the least, prompt rejection of the submitted manuscript should be expected. If the editor was not aware of the violations and the article has already been published, then a notice of redundant or duplicate publication will probably be published with or without the author's explanation or approval.

Acceptable Secondary Publication

Secondary publication in the same or another language, especially in other countries, is justifiable, and can be beneficial, provided all of the following conditions are met:

- The authors have received approval from the editors of both journals; the editor concerned with secondary publication must have a photocopy, reprint, or manuscript of the primary version.
- The priority of the primary publication is respected by a publication interval of at least one week (unless specifically negotiated otherwise by both editors).
- The paper for secondary publication is intended for a different group of readers; an abbreviated version could be sufficient.
- The secondary version reflects faithfully the data and interpretations of the primary version.
- A footnote on the title page of the secondary version informs readers, peers, and documenting agencies that the paper has been published in whole or in part and states the primary reference. A suitable footnote might read: "This article is based on a study first reported in the [title of journal, with full reference]."

Conflict of Interest

Authors must indicate whether or not they have a financial relationship with an organization that sponsored the research. They should also state that they have full control of all primary data and that they agree to allow the journal to review their data if requested.

Reproduced from: International Committee of Medical Journal Editors. Uniform Requirements for Manuscripts Submitted to Biomedical Journals. http://www.icmje.org/

Certificate of Exclusive Submission and Disclosure of Conflict of Interest

ID: JG-

The manuscript entitled: ....

....

....

is original. The author(s) hereby certifies(-fy) that none of the material in this manuscript has been or will be published and none is currently under consideration for publication elsewhere, and that the Conflict of Interest Disclosure Statement on the ScholarOne Manuscript was completed at the time of submission.

Date ..31/10/16..... Full name (printed) ..Richard Parker........ Signature..........................

Date ....17/02/2017........ Full name (printed) ANTONELLA GHEZZI Signature..........................

Date .... Full name (printed) .... Signature..........................

Date .... Full name (printed) .... Signature..........................

Date .... Full name (printed) .... Signature..........................

Date .... Full name (printed) .... Signature..........................

Date .... Full name (printed) .... Signature..........................

Date .... Full name (printed) .... Signature..........................

Date .... Full name (printed) .... Signature..........................

Date .... Full name (printed) .... Signature..........................

The certificate must be signed by all authors.

Redundant or Duplicate Publication

When submitting a paper, an author should always make a full statement to the editor about all submissions and previous reports that might be regarded as redundant or duplicate publication of the same or very similar work. The author should alert the editor if the work includes subjects about whom a previous report has been published. Any such work should be referred to and referenced in the new paper. Copies of such material should be included with the submitted paper to help the editor decide how to deal with the matter.

If redundant or duplicate publication is attempted or occurs without such notification, authors should expect editorial action to be taken. At the least, prompt rejection of the submitted manuscript should be expected. If the editor was not aware of the violations and the article has already been published, then a notice of redundant or duplicate publication will probably be published with or without the author's explanation or approval.

Acceptable Secondary Publication

Secondary publication in the same or another language, especially in other countries, is justifiable, and can be beneficial, provided all of the following conditions are met:

- The authors have received approval from the editors of both journals; the editor concerned with secondary publication must have a photocopy, reprint, or manuscript of the primary version.
- The priority of the primary publication is respected by a publication interval of at least one week (unless specifically negotiated otherwise by both editors).
- The paper for secondary publication is intended for a different group of readers; an abbreviated version could be sufficient.
- The secondary version reflects faithfully the data and interpretations of the primary version.
- A footnote on the title page of the secondary version informs readers, peers, and documenting agencies that the paper has been published in whole or in part and states the primary reference. A suitable footnote might read: "This article is based on a study first reported in the [title of journal, with full reference]."

Conflict of Interest

Authors must indicate whether or not they have a financial relationship with an organization that sponsored the research. They should also state that they have full control of all primary data and that they agree to allow the journal to review their data if requested.

Reproduced from: International Committee of Medical Journal Editors. Uniform Requirements for Manuscripts Submitted to Biomedical Journals
http://www.icmje.org/

Certificate of Exclusive Submission and Disclosure of Conflict of Interest

ID: JG-

The manuscript entitled: ....

is original. The author(s) hereby certifies(-fy) that none of the material in this manuscript has been or will be published and none is currently under consideration for publication elsewhere, and that the Conflict of Interest Disclosure Statement on the ScholarOne Manuscript was completed at the time of submission.

Date 31/10/16 Full name (printed) Richard Parker Signature ....

Date 20/02/17 Full name (printed) ASHISH SINHA Signature Ashish Sinha

Date .... Full name (printed) .... Signature ....

Date .... Full name (printed) .... Signature ....

Date .... Full name (printed) .... Signature ....

Date .... Full name (printed) .... Signature ....

Date .... Full name (printed) .... Signature ....

Date .... Full name (printed) .... Signature ....

Date .... Full name (printed) .... Signature ....

Date .... Full name (printed) .... Signature ....

The certificate must be signed by all authors.

Scanned by CamScanner

Redundant or Duplicate Publication

When submitting a paper, an author should always make a full statement to the editor about all submissions and previous reports that might be regarded as redundant or duplicate publication of the same or very similar work. The author should alert the editor if the work includes subjects about whom a previous report has been published. Any such work should be referred to and referenced in the new paper. Copies of such material should be included with the submitted paper to help the editor decide how to deal with the matter.

If redundant or duplicate publication is attempted or occurs without such notification, authors should expect editorial action to be taken. At the least, prompt rejection of the submitted manuscript should be expected. If the editor was not aware of the violations and the article has already been published, then a notice of redundant or duplicate publication will probably be published with or without the author's explanation or approval.

Acceptable Secondary Publication

Secondary publication in the same or another language, especially in other countries, is justifiable, and can be beneficial, provided all of the following conditions are met:

- The authors have received approval from the editors of both journals; the editor concerned with secondary publication must have a photocopy, reprint, or manuscript of the primary version.
- The priority of the primary publication is respected by a publication interval of at least one week (unless specifically negotiated otherwise by both editors).
- The paper for secondary publication is intended for a different group of readers; an abbreviated version could be sufficient.
- The secondary version reflects faithfully the data and interpretations of the primary version.
- A footnote on the title page of the secondary version informs readers, peers, and documenting agencies that the paper has been published in whole or in part and states the primary reference. A suitable footnote might read: "This article is based on a study first reported in the [title of journal, with full reference]."

Conflict of Interest

Authors must indicate whether or not they have a financial relationship with an organization that sponsored the research. They should also state that they have full control of all primary data and that they agree to allow the journal to review their data if requested.

Reproduced from: International Committee of Medical Journal Editors. Uniform Requirements for Manuscripts Submitted to Biomedical Journals. http://www.icmje.org/

Certificate of Exclusive Submission and Disclosure of Conflict of Interest

ID: JG-

The manuscript entitled: Clinical and Microbiological Features of Infection in Alcoholic Hepatitis: An International Cohort Study

is original. The author(s) hereby certifies(-fy) that none of the material in this manuscript has been or will be published and none is currently under consideration for publication elsewhere, and that the Conflict of Interest Disclosure Statement on the ScholarOne Manuscript was completed at the time of submission.

Date 16/1/2017 Full name (printed) RICHARD PARKER Signature

Date 18/01/2017 Full name (printed) ANDY HOLT Signature

Date 3/2/2017 Full name (printed) MIRUNA DAVID Signature

Date .... Full name (printed) .... Signature ....

Date .... Full name (printed) .... Signature ....

Date .... Full name (printed) .... Signature ....

Date .... Full name (printed) .... Signature ....

Date .... Full name (printed) .... Signature ....

Date .... Full name (printed) .... Signature ....

Date .... Full name (printed) .... Signature ....

The certificate must be signed by all authors.

Scanned by CamScanner

Redundant or Duplicate Publication

When submitting a paper, an author should always make a full statement to the editor about all submissions and previous reports that might be regarded as redundant or duplicate publication of the same or very similar work. The author should alert the editor if the work includes subjects about whom a previous report has been published. Any such work should be referred to and referenced in the new paper. Copies of such material should be included with the submitted paper to help the editor decide how to deal with the matter.

If redundant or duplicate publication is attempted or occurs without such notification, authors should expect editorial action to be taken. At the least, prompt rejection of the submitted manuscript should be expected. If the editor was not aware of the violations and the article has already been published, then a notice of redundant or duplicate publication will probably be published with or without the author's explanation or approval.

Acceptable Secondary Publication

Secondary publication in the same or another language, especially in other countries, is justifiable, and can be beneficial, provided all of the following conditions are met:

- The authors have received approval from the editors of both journals; the editor concerned with secondary publication must have a photocopy, reprint, or manuscript of the primary version.
- The priority of the primary publication is respected by a publication interval of at least one week (unless specifically negotiated otherwise by both editors).
- The paper for secondary publication is intended for a different group of readers; an abbreviated version could be sufficient.
- The secondary version reflects faithfully the data and interpretations of the primary version.
- A footnote on the title page of the secondary version informs readers, peers, and documenting agencies that the paper has been published in whole or in part and states the primary reference. A suitable footnote might read: "This article is based on a study first reported in the [title of journal, with full reference]."

Conflict of Interest

Authors must indicate whether or not they have a financial relationship with an organization that sponsored the research. They should also state that they have full control of all primary data and that they agree to allow the journal to review their data if requested.

Reproduced from: International Committee of Medical Journal Editors. Uniform Requirements for Manuscripts Submitted to Biomedical Journals. http://www.icmje.org/

Certificate of Exclusive Submission and Disclosure of Conflict of Interest

ID: JG-

The manuscript entitled: ....

....

is original. The author(s) hereby certifies(-fy) that none of the material in this manuscript has been or will be published and none is currently under consideration for publication elsewhere, and that the Conflict of Interest Disclosure Statement on the ScholarOne Manuscript was completed at the time of submission.

Date 20/01/17 .... Full name (printed) María José Sánchez Pérez .... Signature ....

Date 20/01/17 .... Full name (printed) Emilio González Reimers .... Signature ....

Date 20/01/2017 .... Full name (printed) Onán Pérez Hernández .... Signature ....

Date .... Full name (printed) .... Signature ....

Date .... Full name (printed) .... Signature ....

Date .... Full name (printed) .... Signature ....

Date .... Full name (printed) .... Signature ....

Date .... Full name (printed) .... Signature ....

Date .... Full name (printed) .... Signature ....

Date .... Full name (printed) .... Signature ....

The certificate must be signed by all authors.

Scanned by CamScanner

Redundant or Duplicate Publication

When submitting a paper, an author should always make a full statement to the editor about all submissions and previous reports that might be regarded as redundant or duplicate publication of the same or very similar work. The author should alert the editor if the work includes subjects about whom a previous report has been published. Any such work should be referred to and referenced in the new paper. Copies of such material should be included with the submitted paper to help the editor decide how to deal with the matter.

If redundant or duplicate publication is attempted or occurs without such notification, authors should expect editorial action to be taken. At the least, prompt rejection of the submitted manuscript should be expected. If the editor was not aware of the violations and the article has already been published, then a notice of redundant or duplicate publication will probably be published with or without the author's explanation or approval.

Acceptable Secondary Publication

Secondary publication in the same or another language, especially in other countries, is justifiable, and can be beneficial, provided all of the following conditions are met:

- The authors have received approval from the editors of both journals; the editor concerned with secondary publication must have a photocopy, reprint, or manuscript of the primary version.
- The priority of the primary publication is respected by a publication interval of at least one week (unless specifically negotiated otherwise by both editors).
- The paper for secondary publication is intended for a different group of readers; an abbreviated version could be sufficient.
- The secondary version reflects faithfully the data and interpretations of the primary version.
- A footnote on the title page of the secondary version informs readers, peers, and documenting agencies that the paper has been published in whole or in part and states the primary reference. A suitable footnote might read: "This article is based on a study first reported in the [title of journal, with full reference]."

Conflict of Interest

Authors must indicate whether or not they have a financial relationship with an organization that sponsored the research. They should also state that they have full control of all primary data and that they agree to allow the journal to review their data if requested.

Reproduced from: International Committee of Medical Journal Editors. Uniform Requirements for Manuscripts Submitted to Biomedical Journals. http://www.icmje.org/

Certificate of Exclusive Submission and Disclosure of Conflict of Interest

ID: JG-

The manuscript entitled: ....

....

....

is original. The author(s) hereby certifies(-fy) that none of the material in this manuscript has been or will be published and none is currently under consideration for publication elsewhere, and that the Conflict of Interest Disclosure Statement on the ScholarOne Manuscript was completed at the time of submission.

Date 31/10/16 ..... Full name (printed) Richard Parker ..... Signature ....

Date 01/02/17 ..... Full name (printed) ADITI KUMAR ..... Signature ....

Date .... Full name (printed) .... Signature ....

Date .... Full name (printed) .... Signature ....

Date .... Full name (printed) .... Signature ....

Date .... Full name (printed) .... Signature ....

Date .... Full name (printed) .... Signature ....

Date .... Full name (printed) .... Signature ....

Date .... Full name (printed) .... Signature ....

Date .... Full name (printed) .... Signature ....

The certificate must be signed by all authors.

Redundant or Duplicate Publication

When submitting a paper, an author should always make a full statement to the editor about all submissions and previous reports that might be regarded as redundant or duplicate publication of the same or very similar work. The author should alert the editor if the work includes subjects about whom a previous report has been published. Any such work should be referred to and referenced in the new paper. Copies of such material should be included with the submitted paper to help the editor decide how to deal with the matter.

If redundant or duplicate publication is attempted or occurs without such notification, authors should expect editorial action to be taken. At the least, prompt rejection of the submitted manuscript should be expected. If the editor was not aware of the violations and the article has already been published, then a notice of redundant or duplicate publication will probably be published with or without the author's explanation or approval.

Acceptable Secondary Publication

Secondary publication in the same or another language, especially in other countries, is justifiable, and can be beneficial, provided all of the following conditions are met:

- The authors have received approval from the editors of both journals; the editor concerned with secondary publication must have a photocopy, reprint, or manuscript of the primary version.
- The priority of the primary publication is respected by a publication interval of at least one week (unless specifically negotiated otherwise by both editors).
- The paper for secondary publication is intended for a different group of readers; an abbreviated version could be sufficient.
- The secondary version reflects faithfully the data and interpretations of the primary version.
- A footnote on the title page of the secondary version informs readers, peers, and documenting agencies that the paper has been published in whole or in part and states the primary reference. A suitable footnote might read: "This article is based on a study first reported in the [title of journal, with full reference]."

Conflict of Interest

Authors must indicate whether or not they have a financial relationship with an organization that sponsored the research. They should also state that they have full control of all primary data and that they agree to allow the journal to review their data if requested.

Reproduced from: International Committee of Medical Journal Editors. Uniform Requirements for Manuscripts Submitted to Biomedical Journals. http://www.icmje.org/

Certificate of Exclusive Submission and Disclosure of Conflict of Interest

ID: JG-

The manuscript entitled: CLINICAL + MICROBIOLOGICAL FEATURES OF INFECTION IN ALCOHOLIC HEPATITIS: AN INTERNATIONAL COHORT STUDY.

is original. The author(s) hereby certifies(-fy) that none of the material in this manuscript has been or will be published and none is currently under consideration for publication elsewhere, and that the Conflict of Interest Disclosure Statement on the ScholarOne Manuscript was completed at the time of submission.

Date 31/10/16 Full name (printed) Richard Parker Signature

Date 18/1/17 Full name (printed) ANNE McCUNE Signature AMcCune.

Date Full name (printed) Signature

Date Full name (printed) Signature

Date Full name (printed) Signature

Date Full name (printed) Signature

Date Full name (printed) Signature

Date Full name (printed) Signature

Date Full name (printed) Signature

Date Full name (printed) Signature

The certificate must be signed by all authors.

Redundant or Duplicate Publication

When submitting a paper, an author should always make a full statement to the editor about all submissions and previous reports that might be regarded as redundant or duplicate publication of the same or very similar work. The author should alert the editor if the work includes subjects about whom a previous report has been published. Any such work should be referred to and referenced in the new paper. Copies of such material should be included with the submitted paper to help the editor decide how to deal with the matter.

If redundant or duplicate publication is attempted or occurs without such notification, authors should expect editorial action to be taken. At the least, prompt rejection of the submitted manuscript should be expected. If the editor was not aware of the violations and the article has already been published, then a notice of redundant or duplicate publication will probably be published with or without the author's explanation or approval.

Acceptable Secondary Publication

Secondary publication in the same or another language, especially in other countries, is justifiable, and can be beneficial, provided all of the following conditions are met:

- The authors have received approval from the editors of both journals; the editor concerned with secondary publication must have a photocopy, reprint, or manuscript of the primary version.
- The priority of the primary publication is respected by a publication interval of at least one week (unless specifically negotiated otherwise by both editors).
- The paper for secondary publication is intended for a different group of readers; an abbreviated version could be sufficient.
- The secondary version reflects faithfully the data and interpretations of the primary version.
- A footnote on the title page of the secondary version informs readers, peers, and documenting agencies that the paper has been published in whole or in part and states the primary reference. A suitable footnote might read: "This article is based on a study first reported in the [title of journal, with full reference]."

Conflict of Interest

Authors must indicate whether or not they have a financial relationship with an organization that sponsored the research. They should also state that they have full control of all primary data and that they agree to allow the journal to review their data if requested.

Reproduced from: International Committee of Medical Journal Editors. Uniform Requirements for Manuscripts Submitted to Biomedical Journals. http://www.icmje.org/

Certificate of Exclusive Submission and Disclosure of Conflict of Interest

ID: JG-

The manuscript entitled: ....
....
....
is original. The author(s) hereby certifies(-fy) that none of the material in this manuscript has been or will be published and none is currently under consideration for publication elsewhere, and that the Conflict of Interest Disclosure Statement on the ScholarOne Manuscript was completed at the time of submission.

Date .... Full name (printed) .... Signature ....

Date 17/01/17 .... Full name (printed) Guruprasad Aithal .... Signature ....

Date .... Full name (printed) .... Signature ....

Date .... Full name (printed) .... Signature ....

Date .... Full name (printed) .... Signature ....

Date .... Full name (printed) .... Signature ....

Date .... Full name (printed) .... Signature ....

Date .... Full name (printed) .... Signature ....

Date .... Full name (printed) .... Signature ....

Date .... Full name (printed) .... Signature ....

The certificate must be signed by all authors.

Redundant or Duplicate Publication

When submitting a paper, an author should always make a full statement to the editor about all submissions and previous reports that might be regarded as redundant or duplicate publication of the same or very similar work. The author should alert the editor if the work includes subjects about whom a previous report has been published. Any such work should be referred to and referenced in the new paper. Copies of such material should be included with the submitted paper to help the editor decide how to deal with the matter.

If redundant or duplicate publication is attempted or occurs without such notification, authors should expect editorial action to be taken. At the least, prompt rejection of the submitted manuscript should be expected. If the editor was not aware of the violations and the article has already been published, then a notice of redundant or duplicate publication will probably be published with or without the author's explanation or approval.

Acceptable Secondary Publication

Secondary publication in the same or another language, especially in other countries, is justifiable, and can be beneficial, provided all of the following conditions are met:

- The authors have received approval from the editors of both journals; the editor concerned with secondary publication must have a photocopy, reprint, or manuscript of the primary version.
- The priority of the primary publication is respected by a publication interval of at least one week (unless specifically negotiated otherwise by both editors).
- The paper for secondary publication is intended for a different group of readers; an abbreviated version could be sufficient.
- The secondary version reflects faithfully the data and interpretations of the primary version.
- A footnote on the title page of the secondary version informs readers, peers, and documenting agencies that the paper has been published in whole or in part and states the primary reference. A suitable footnote might read: "This article is based on a study first reported in the [title of journal, with full reference]."

Conflict of Interest

Authors must indicate whether or not they have a financial relationship with an organization that sponsored the research. They should also state that they have full control of all primary data and that they agree to allow the journal to review their data if requested.

Reproduced from: International Committee of Medical Journal Editors. Uniform Requirements for Manuscripts Submitted to Biomedical Journals. http://www.icmje.org/

Certificate of Exclusive Submission and Disclosure of Conflict of Interest

ID: JG-

The manuscript entitled: ....
....
....
is original. The author(s) hereby certifies(-fy) that none of the material in this manuscript has been or will be published and none is currently under consideration for publication elsewhere, and that the Conflict of Interest Disclosure Statement on the ScholarOne Manuscript was completed at the time of submission.

Date 31/10/16 Full name (printed) Richard Parker Signature ....

Date 17/01/2017 Full name (printed) Chris Corbett Signature ....

Date .... Full name (printed) .... Signature ....

Date .... Full name (printed) .... Signature ....

Date .... Full name (printed) .... Signature ....

Date .... Full name (printed) .... Signature ....

Date .... Full name (printed) .... Signature ....

Date .... Full name (printed) .... Signature ....

Date .... Full name (printed) .... Signature ....

Date .... Full name (printed) .... Signature ....

The certificate must be signed by all authors.

Redundant or Duplicate Publication

When submitting a paper, an author should always make a full statement to the editor about all submissions and previous reports that might be regarded as redundant or duplicate publication of the same or very similar work. The author should alert the editor if the work includes subjects about whom a previous report has been published. Any such work should be referred to and referenced in the new paper. Copies of such material should be included with the submitted paper to help the editor decide how to deal with the matter.

If redundant or duplicate publication is attempted or occurs without such notification, authors should expect editorial action to be taken. At the least, prompt rejection of the submitted manuscript should be expected. If the editor was not aware of the violations and the article has already been published, then a notice of redundant or duplicate publication will probably be published with or without the author's explanation or approval.

Acceptable Secondary Publication

Secondary publication in the same or another language, especially in other countries, is justifiable, and can be beneficial, provided all of the following conditions are met:

- The authors have received approval from the editors of both journals; the editor concerned with secondary publication must have a photocopy, reprint, or manuscript of the primary version.
- The priority of the primary publication is respected by a publication interval of at least one week (unless specifically negotiated otherwise by both editors).
- The paper for secondary publication is intended for a different group of readers; an abbreviated version could be sufficient.
- The secondary version reflects faithfully the data and interpretations of the primary version.
- A footnote on the title page of the secondary version informs readers, peers, and documenting agencies that the paper has been published in whole or in part and states the primary reference. A suitable footnote might read: "This article is based on a study first reported in the [title of journal, with full reference]."

Conflict of Interest

Authors must indicate whether or not they have a financial relationship with an organization that sponsored the research. They should also state that they have full control of all primary data and that they agree to allow the journal to review their data if requested.

Reproduced from: International Committee of Medical Journal Editors. Uniform Requirements for Manuscripts Submitted to Biomedical Journals. http://www.icmje.org/

Certificate of Exclusive Submission and Disclosure of Conflict of Interest

ID: JG-

The manuscript entitled: ....

....

....

is original. The author(s) hereby certifies(-fy) that none of the material in this manuscript has been or will be published and none is currently under consideration for publication elsewhere, and that the Conflict of Interest Disclosure Statement on the ScholarOne Manuscript was completed at the time of submission.

Date ..31/10/16..... Full name (printed) ..Richard Parker...... Signature..........................

Date .27/1/2017.... Full name (printed) Jonathan Nahas... Signature..........................

Date .... Full name (printed) .... Signature..........................

Date .... Full name (printed) .... Signature..........................

Date .... Full name (printed) .... Signature..........................

Date .... Full name (printed) .... Signature..........................

Date .... Full name (printed) .... Signature..........................

Date .... Full name (printed) .... Signature..........................

Date .... Full name (printed) .... Signature..........................

Date .... Full name (printed) .... Signature..........................

The certificate must be signed by all authors.

[illegible]dundant or Duplicate Publication

[illegible]en submitting a paper, an author should always make a [illegible] statement to the editor about all submissions and previ[illegible] reports that might be regarded as redundant or duplicate [illegible]blication of the same or very similar work. The author [illegible]uld alert the editor if the work includes subjects about [illegible]om a previous report has been published. Any such work [illegible]uld be referred to and referenced in the new paper. [illegible]pies of such material should be included with the submit[illegible] paper to help the editor decide how to deal with the [illegible]tter.

[illegible]f redundant or duplicate publication is attempted or occurs [illegible]hout such notification, authors should expect editorial [illegible]ion to be taken. At the least, prompt rejection of the [illegible]mitted manuscript should be expected. If the editor was [illegible] aware of the violations and the article has already been [illegible]lished, then a notice of redundant or duplicate publication [illegible] probably be published with or without the author's [illegible]lanation or approval.

[illegible]ceptable Secondary Publication

[illegible]ondary publication in the same or another language, espe[illegible]y in other countries, is justifiable, and can be beneficial, [illegible]vided all of the following conditions are met:

- The authors have received approval from the editors of both journals; the editor concerned with secondary publication must have a photocopy, reprint, or manuscript of the primary version.
- The priority of the primary publication is respected by a publication interval of at least one week (unless specifically negotiated otherwise by both editors).
- The paper for secondary publication is intended for a different group of readers; an abbreviated version could be sufficient.
- The secondary version reflects faithfully the data and interpretations of the primary version.
- A footnote on the title page of the secondary version informs readers, peers, and documenting agencies that the paper has been published in whole or in part and states the primary reference. A suitable footnote might read: "This article is based on a study first reported in the [title of journal, with full reference]."

Conflict of Interest

Authors must indicate whether or not they have a financial relationship with an organization that sponsored the research. They should also state that they have full control of all primary data and that they agree to allow the journal to review their data if requested.

[illegible]oduced from: International Committee of Medical Journal Editors. Uniform Requirements for Manuscripts Submitted to Biomedical Journals. [illegible]//www.icmje.org/

[illegible]tificate of Exclusive Submission and Disclosure of Conflict of Interest

ID: JG-

[illegible] manuscript entitled: ....

....

[illegible]riginal. The author(s) hereby certifies(-fy) that none of the material in this manuscript has been or will be [illegible]ished and none is currently under consideration for publication elsewhere, and that the Conflict of Interest Disclosure [illegible]ment on the ScholarOne Manuscript was completed at the time of submission.

31/10/16	Full name (printed)	Richard Parker	Signature
23/1/17	Full name (printed)	Stephen Stewart	Signature [signature]
	Full name (printed)		Signature
	Full name (printed)		Signature
	Full name (printed)		Signature
	Full name (printed)		Signature
	Full name (printed)		Signature
	Full name (printed)		Signature
	Full name (printed)		Signature
	[illegible]		[illegible]

Redundant or Duplicate Publication

When submitting a paper, an author should always make a full statement to the editor about all submissions and previous reports that might be regarded as redundant or duplicate publication of the same or very similar work. The author should alert the editor if the work includes subjects about whom a previous report has been published. Any such work should be referred to and referenced in the new paper. Copies of such material should be included with the submitted paper to help the editor decide how to deal with the matter.

If redundant or duplicate publication is attempted or occurs without such notification, authors should expect editorial action to be taken. At the least, prompt rejection of the submitted manuscript should be expected. If the editor was not aware of the violations and the article has already been published, then a notice of redundant or duplicate publication will probably be published with or without the author's explanation or approval.

Acceptable Secondary Publication

Secondary publication in the same or another language, especially in other countries, is justifiable, and can be beneficial, provided all of the following conditions are met:

- The authors have received approval from the editors of both journals; the editor concerned with secondary publication must have a photocopy, reprint, or manuscript of the primary version.
- The priority of the primary publication is respected by a publication interval of at least one week (unless specifically negotiated otherwise by both editors).
- The paper for secondary publication is intended for a different group of readers; an abbreviated version could be sufficient.
- The secondary version reflects faithfully the data and interpretations of the primary version.
- A footnote on the title page of the secondary version informs readers, peers, and documenting agencies that the paper has been published in whole or in part and states the primary reference. A suitable footnote might read: "This article is based on a study first reported in the [title of journal, with full reference]."

Conflict of Interest

Authors must indicate whether or not they have a financial relationship with an organization that sponsored the research. They should also state that they have full control of all primary data and that they agree to allow the journal to review their data if requested.

Reproduced from: International Committee of Medical Journal Editors. Uniform Requirements for Manuscripts Submitted to Biomedical Journals. http://www.icmje.org/

Certificate of Exclusive Submission and Disclosure of Conflict of Interest

ID: JG-

The manuscript entitled: ....

....

....

is original. The author(s) hereby certifies(-fy) that none of the material in this manuscript has been or will be published and none is currently under consideration for publication elsewhere, and that the Conflict of Interest Disclosure Statement on the ScholarOne Manuscript was completed at the time of submission.

Date ...31/10/16..... Full name (printed) ...Richard Parker..... Signature..........
Date ...30/01/17..... Full name (printed) ...DANIEL J. WHEATLEY..... Signature..........
Date .... Full name (printed) .... Signature..........
Date .... Full name (printed) .... Signature..........
Date .... Full name (printed) .... Signature..........
Date .... Full name (printed) .... Signature..........
Date .... Full name (printed) .... Signature..........
Date .... Full name (printed) .... Signature..........
Date .... Full name (printed) .... Signature..........
Date .... Full name (printed) .... Signature..........

The certificate must be signed by all authors.

Redundant or Duplicate Publication

When submitting a paper, an author should always make a full statement to the editor about all submissions and previous reports that might be regarded as redundant or duplicate publication of the same or very similar work. The author should alert the editor if the work includes subjects about whom a previous report has been published. Any such work should be referred to and referenced in the new paper. Copies of such material should be included with the submitted paper to help the editor decide how to deal with the matter.

If redundant or duplicate publication is attempted or occurs without such notification, authors should expect editorial action to be taken. At the least, prompt rejection of the submitted manuscript should be expected. If the editor was not aware of the violations and the article has already been published, then a notice of redundant or duplicate publication will probably be published with or without the author's explanation or approval.

Acceptable Secondary Publication

Secondary publication in the same or another language, especially in other countries, is justifiable, and can be beneficial, provided all of the following conditions are met:

- The authors have received approval from the editors of both journals; the editor concerned with secondary publication must have a photocopy, reprint, or manuscript of the primary version.
- The priority of the primary publication is respected by a publication interval of at least one week (unless specifically negotiated otherwise by both editors).
- The paper for secondary publication is intended for a different group of readers; an abbreviated version could be sufficient.
- The secondary version reflects faithfully the data and interpretations of the primary version.
- A footnote on the title page of the secondary version informs readers, peers, and documenting agencies that the paper has been published in whole or in part and states the primary reference. A suitable footnote might read: "This article is based on a study first reported in the [title of journal, with full reference]."

Conflict of Interest

Authors must indicate whether or not they have a financial relationship with an organization that sponsored the research. They should also state that they have full control of all primary data and that they agree to allow the journal to review their data if requested.

Reproduced from: International Committee of Medical Journal Editors. Uniform Requirements for Manuscripts Submitted to Biomedical Journals. http://www.icmje.org/

Certificate of Exclusive Submission and Disclosure of Conflict of Interest

ID: JG-

The manuscript entitled: ....
....
....
is original. The author(s) hereby certifies(-fy) that none of the material in this manuscript has been or will be published and none is currently under consideration for publication elsewhere, and that the Conflict of Interest Disclosure Statement on the ScholarOne Manuscript was completed at the time of submission.

Date 31/10/16 Full name (printed) Richard Parker Signature ....
Date 15/02/2017 Full name (printed) Fiona Jones Signature [signature]
Date .... Full name (printed) .... Signature ....
Date .... Full name (printed) .... Signature ....
Date .... Full name (printed) .... Signature ....
Date .... Full name (printed) .... Signature ....
Date .... Full name (printed) .... Signature ....
Date .... Full name (printed) .... Signature ....
Date .... Full name (printed) .... Signature ....
Date .... Full name (printed) .... Signature ....

The certificate must be signed by all authors.

Redundant or Duplicate Publication

When submitting a paper, an author should always make a full statement to the editor about all submissions and previous reports that might be regarded as redundant or duplicate publication of the same or very similar work. The author should alert the editor if the work includes subjects about whom a previous report has been published. Any such work should be referred to and referenced in the new paper. Copies of such material should be included with the submitted paper to help the editor decide how to deal with the matter.

If redundant or duplicate publication is attempted or occurs without such notification, authors should expect editorial action to be taken. At the least, prompt rejection of the submitted manuscript should be expected. If the editor was not aware of the violations and the article has already been published, then a notice of redundant or duplicate publication will probably be published with or without the author's explanation or approval.

Acceptable Secondary Publication

Secondary publication in the same or another language, especially in other countries, is justifiable, and can be beneficial, provided all of the following conditions are met:

- The authors have received approval from the editors of both journals; the editor concerned with secondary publication must have a photocopy, reprint, or manuscript of the primary version.
- The priority of the primary publication is respected by a publication interval of at least one week (unless specifically negotiated otherwise by both editors).
- The paper for secondary publication is intended for a different group of readers; an abbreviated version could be sufficient.
- The secondary version reflects faithfully the data and interpretations of the primary version.
- A footnote on the title page of the secondary version informs readers, peers, and documenting agencies that the paper has been published in whole or in part and states the primary reference. A suitable footnote might read: "This article is based on a study first reported in the [title of journal, with full reference]."

Conflict of Interest

Authors must indicate whether or not they have a financial relationship with an organization that sponsored the research. They should also state that they have full control of all primary data and that they agree to allow the journal to review their data if requested.

Reproduced from: International Committee of Medical Journal Editors, Uniform Requirements for Manuscripts Submitted to Biomedical Journals. http://www.icmje.org/

Certificate of Exclusive Submission and Disclosure of Conflict of Interest

ID: JG-

The manuscript entitled: ....

is original. The author(s) hereby certifies(-fy) that none of the material in this manuscript has been or will be published and none is currently under consideration for publication elsewhere, and that the Conflict of Interest Disclosure Statement on the ScholarOne Manuscript was completed at the time of submission.

Date 31/10/16 Full name (printed) Richard Parker Signature ....
Date 02/02/2017 Full name (printed) Gene Im Signature [signature]
Date .... Full name (printed) .... Signature ....
Date .... Full name (printed) .... Signature ....
Date .... Full name (printed) .... Signature ....
Date .... Full name (printed) .... Signature ....
Date .... Full name (printed) .... Signature ....
Date .... Full name (printed) .... Signature ....
Date .... Full name (printed) .... Signature ....
Date .... Full name (printed) .... Signature ....

The certificate must be signed by all authors.
